# Supplementary material for: HIV knowledge and reported stigma among South Florida health fair participants
Source: PLoS One. 2025 Aug 19;20(8):e0329665. doi: 10.1371/journal.pone.0329665 (PMC12364354; doi:10.1371/journal.pone.0329665)
Supplement: S1 Appendix — (DOCX) [file pone.0329665.s001.docx]

**S1 Appendix**

Please check either the yes or no box that best represents your own knowledge and attitudes. Some questions will ask you to select all that apply. For these questions, please select all the responses that best represent your own knowledge and attitudes.

**Demographics**

1. What is your age (select one)?
   1. 17 or younger
   2. 18-20
   3. 21-29
   4. 30-39
   5. 40-49
   6. 50-59
   7. 60-75
   8. Over 75
2. What is your self-identified gender?
    a. Male

b. Female

c. Other _____

1. What is your self-identified race (Check all that apply)?
   1. American Indian or Alaska Native
   2. Asian
   3. Black or African American
   4. Hispanic/Latino
   5. Native Hawaiian or Pacific Islander
   6. White/Caucasian
   7. Other _____
2. What language do you primarily speak at home (Check all that apply)?
   1. Haitian Creole
   2. English
   3. Spanish
   4. Other _____
3. What is your highest level of education?
   1. Some high school
   2. Highschool
   3. Some college
   4. College
   5. Grad school/professional school
   6. Other _____
4. What is your country of origin?
   1. USA
   2. Cuba
   3. Haiti
   4. Colombia

**HIV Testing Attitude**

7. I was nervous to get HIV tested today (yes or no).

8. If yes, I was nervous because **(check all that apply)**

- - I am fearful of testing positive.
  - I am worried that my results would not be kept confidential.
  - I am unaware of available HIV treatments.
  - I do not believe that I could afford HIV treatments, if I tested positive.
  - I am worried about disclosing my HIV status, if I tested positive.
  - Other ______

**Transmission Knowledge**

9. HIV can be spread through kissing.

10. HIV can be spread by sharing needles for injection drugs.

11. HIV can be spread through mosquito bites.

12. HIV can be spread by drinking from the same glass as a person with HIV.

13.HIV can be spread through condomless anal sex.

14.HIV can be spread through condomless vaginal sex.

15. People with HIV have a shortened lifespan, even if they receive anti-viral treatment.

16. I have heard of post-exposure prophylaxis (PEP).

17. I have heard of pre-exposure prophylaxis (PREP).

**General Knowledge**

18. How have you learned about HIV? **(Check all that apply)**

- - Doctors/ healthcare professionals
  - Past DOCS Health Fair
  - Internet/ social media
  - School
  - Family/friends
  - TV shows/movies
  - News
  - Public health agencies
  - Other: ______

**Stigma**

19. I have respect for people living with HIV.

20. I believe that people living with HIV deserve their situation.

21. People talk badly about people living with HIV to others.

22. I am worried about what my family or friends would think of me if I tested positive.

**Perceived Risk**

23. I believe I am at risk of contracting HIV.

23a. If so, why? (Select all that apply)

- I received a blood transfusion before 1992.
- I have had unprotected sex with more than one partner in the past year.
- Men only: I have sex with other men.
- I use IV drugs.
- None of the above apply to me
- Other _____
