# Supplementary material for: HIV knowledge and reported stigma among South Florida health fair participants
Source: PLoS One. 2025 Aug 19;20(8):e0329665. doi: 10.1371/journal.pone.0329665 (PMC12364354; doi:10.1371/journal.pone.0329665)
Supplement: Attachment — Submitted filename: HIV Knowledge.docx [file pone.0329665.s003.docx]

**Overall, this is a well-structured, impactful study** that contributes valuable insights into HIV knowledge and stigma. The **main areas for improvement** are **clarity, conciseness, and stronger calls to action in the discussion and conclusion.**

**Wordiness & Redundancy**
Some sentences are **long and repetitive**, making them harder to read.

**Example:**
"Many of these misconceptions can be traced back to the spread of misinformation during the AIDS epidemic in the 1980s, when little was known about the virus or its transmission."

**Suggested Edit:**
"Many misconceptions stem from misinformation spread during the 1980s AIDS epidemic when the virus was poorly understood."

**Passive Voice Usage**
The manuscript frequently uses **passive voice**, which can make statements less direct and engaging.

**Example:**
"Survey data was analyzed using chi-square analysis at an alpha=0.05."

**Suggested Edit:**
"We analyzed survey data using chi-square tests with an alpha level of 0.05."

**Ambiguity in Some Statements**

**Example:**
"Participants were classified as White non-Hispanic, White Hispanic, or Black as only two participants identified as Black Hispanic and were classified as mixed race."

**Suggested Edit:**
"Participants were categorized as White non-Hispanic, White Hispanic, or Black. Only two participants identified as Black Hispanic and were classified as mixed race."

**Brevity and Clarity:**

**Example:**

*Many of these misconceptions can be traced back to the spread of misinformation during the AIDS epidemic in the 1980s, when little was known about the virus or its transmission.*

**Suggested Edit:**

*Many misconceptions stem from misinformation spread during the 1980s AIDS epidemic when the virus was poorly understood.*

**Original:**

*Among 173 total respondents, 149 received HIV testing and 24 declined HIV testing.*

**Edited:**

*Of the 173 respondents, 149 underwent HIV testing, while 24 declined.*

**Recommendations:**

Use **“prevention” instead of “preventative”** (more common in scientific writing).

Introduce abbreviations **when first mentioned** and use them consistently i.e. PLWH
