# Supplementary material for: HIV knowledge and reported stigma among South Florida health fair participants
Source: PLoS One. 2025 Aug 19;20(8):e0329665. doi: 10.1371/journal.pone.0329665 (PMC12364354; doi:10.1371/journal.pone.0329665)
Supplement: Attachment — Submitted filename: Response to reviews_HIV_final (1).docx [file pone.0329665.s004.docx]

May 8th, 2025

To: The Editor

Dear Dr. Regencia,

Thank you and the reviewers for your insightful review of our manuscript for publication in *PLOS One*. We have carefully considered this feedback and made changes to the manuscript. Below, we have addressed each comment and direct the reviewer to where they have been incorporated. Please update the funding statement as: “Dr. Sternberg is funded by the National Center for Advancing Translational Sciences, award number KL2TR002737.” Upon further consideration, we have shared de-identified data to promote additional research efforts and have uploaded the data to a repository (link below). <http://datadryad.org/share/8EvolkNA6a7g6L5s5UcxP8MR9lHfll3O7UWuyXGF6GM>

Sincerely,

Candice A. Sternberg, MD

Assistant Professor, Dept. of Medicine

University of Miami Miller School of Medicine

1120 NW 14^th^ Street #858

Miami, Florida 33136

Phone: (305) 243- 2398

Email: [c.aurelus@miami.edu](mailto:c.aurelus@miami.edu)

**Editor:**

1. **Please ensure that your manuscript meets PLOS ONE's style requirements, including those for file naming. The PLOS ONE style templates can be found at**

**https://journals.plos.org/plosone/s/file?id=wjVg/PLOSOne_formatting_sample_main_body.pdf** [**https://journals.plos.org/plosone/s/file?id=ba62/PLOSOne_formatting_sample_title_authors_affiliations.pdf**](https://journals.plos.org/plosone/s/file?id=ba62/PLOSOne_formatting_sample_title_authors_affiliations.pdf)

We have ensured that the manuscript complies with style requirements.

2. **Before we proceed with your manuscript, please address the following prompts:**
   a. **If there are ethical or legal restrictions on sharing a de-identified data set, please explain them in detail (e.g., data contain potentially identifying or sensitive patient information, data are owned by a third-party organization, etc.) and who has imposed them (e.g., a Research Ethics Committee or Institutional Review Board, etc.). Please also provide contact information for a data access committee, ethics committee, or other institutional body to which data requests may be sent.**

There are no restrictions on sharing de-identified data.

b. **If there are no restrictions, please upload the minimal anonymized data set necessary to replicate your study findings to a stable, public repository and provide us with the relevant URLs, DOIs, or accession numbers. Please see http://www.bmj.com/content/340/bmj.c181.long for guidelines on how to de-identify and prepare clinical data for publication. For a list of recommended repositories, please see https://journals.plos.org/plosone/s/recommended-repositories. You also have the option of uploading the data as Supporting Information files, but we would recommend depositing data directly to a data repository if possible.**

The data has been uploaded to a repository with the link: [http://datadryad.org/share/8EvolkNA6a7g6L5s5UcxP8MR9lHfll3O7UWuyXGF6GM](https://urldefense.com/v3/__http:/h52wvrds.r.us-west-2.awstrack.me/L0/http:*2F*2Fdatadryad.org*2Fshare*2F8EvolkNA6a7g6L5s5UcxP8MR9lHfll3O7UWuyXGF6GM/1/01010196645f2c26-ba180bed-1c52-4565-b404-bd5a93cbee7e-000000/0qTlw8wWPxJMaY4hoqKNsBSPL_4=424__;JSUlJQ!!KVu0SnhVq1hAFvslES2Y!IbDqcYQTqNC1q5GRETsRplhym0Jl3oPwADN3VTfp1y6b-9P4qesdw66Vl8cbTgILfUwmVKRrib1TvdWBqGBl90oXBr8GsVw$)

c. **Please update your Data Availability statement in the submission form accordingly.**

The data availability statement has been updated.

3. **In the online submission form, you indicated that your data is available only on request from a third party. Please note that your Data Availability Statement is currently missing [the name of the third party contact or institution / contact details for the third party, such as an email address or a link to where data requests can be made]. Please update your statement with the missing information.**

Upon reconsideration, we have decided to publicly share de-identified data. We have updated the online forms.

4. **Please remove any funding-related text from the manuscript and let us know how you would like to update your Funding Statement. Currently, your Funding Statement reads as follows: “Dr. Sternberg is funded by the National Center for Advancing Translational Sciences, award number KL2TR002737.”Please include your amended statements within your cover letter; we will change the online submission form on your behalf.**

Thank you. Funding information was removed from text and updated in the cover letter as requested.

**Reviewer 1:**

Thank you for your review. Your comments have greatly improved the manuscript.

**General comments:**

1. **The authors’ account for the unavailability of the data appears limited. While they mention that the data are available upon request, they do not provide sufficient details about the specific restrictions or exceptional circumstances that prevent public sharing.**

Upon reconsideration, we have decided to publicly share de-identified data to this link: [http://datadryad.org/share/8EvolkNA6a7g6L5s5UcxP8MR9lHfll3O7UWuyXGF6GM](https://urldefense.com/v3/__http:/h52wvrds.r.us-west-2.awstrack.me/L0/http:*2F*2Fdatadryad.org*2Fshare*2F8EvolkNA6a7g6L5s5UcxP8MR9lHfll3O7UWuyXGF6GM/1/01010196645f2c26-ba180bed-1c52-4565-b404-bd5a93cbee7e-000000/0qTlw8wWPxJMaY4hoqKNsBSPL_4=424__;JSUlJQ!!KVu0SnhVq1hAFvslES2Y!IbDqcYQTqNC1q5GRETsRplhym0Jl3oPwADN3VTfp1y6b-9P4qesdw66Vl8cbTgILfUwmVKRrib1TvdWBqGBl90oXBr8GsVw$)

2. **(Page 6, Line 119) “Materials and Methods:” All level 1-3 headings must be written in sentence case with only first word in capital letters.**

Thank you. This has been corrected.

**Abstract:**

1. **(Page 2, Line 30) The phrase “third greatest” should be written in words rather than using numerals (“3rd”) for formality.**

This was clarified (page 2, line 30).

2. **(Page 2, line 30-33) The background section of an abstract must incorporate aim to clarify specific purpose and direction of the study.**

Thank you for this suggestion. We incorporated a sentence to describe the study aims that reads: “This cross-sectional study aimed to assess HIV education and perceptions among diverse populations in South Florida to enhance public health community outreach efforts. Specifically, it investigated how HIV knowledge and perceptions vary based on race, primary language, and country of origin.” (page 2, lines 31-34).

3. **(Page 2, line 34-37) Under Material and methods section, authors should briefly outline the approach, design, sampling method, and tool used for analyses, despite the word limit of the abstract section.**

Thank you for the suggestion. This section now reads: “Cross-sectional surveys were administered at five South Florida health fair locations to evaluate understanding of HIV transmission, strategies for prevention and treatment, and stigma among those who accepted and declined free HIV testing.” (Page 2, lines 35-37).

4. **(Page 2 Line 39) Change “For all respondents” since this is not a general statement but it a comparing from a group of participants, thus the use “Out of all respondents” would best fit this context.**

Thank you, this has been corrected (page 2, line 40).

5. **(Page 2 Line 40) Provide statistics from empirical data for this statement “Over half of all respondents were foreign born”.**

The sentence now reads: “Over half of all respondents were foreign born (59%)” (page 2, line 41).

6. **(Page 2, line 42) Authors must ensure consistent formatting by placing all percentages within parentheses.**

Thank you for the suggestion. The sentence has been rewritten: “Most participants knew HIV can be spread by injection drug usage (98.8%) and unprotected sex (97.7%) (page 2, line 41-43).

7. **(Page 2, Line 44 & line 46-47) If these were results emanated from Chi-square analysis, author should include chi-square statistic value i.e., χ²(2, N = 173) = ….. p = .012.**

Thank you for the suggestion. The chi-square statistic values are now reported in the abstract (page 2, lines 45, 48).

**Introduction:**

1. **(Page 3, Line 59-60) clarify where “Deep South” is**

The phrase was updated to “South” to better reflect the reference (page 4, line 57).

2. **(Page 3, line 62) Write “3rd” in words such “Third” and include statistics that are indicative of a high prevalence of new HIV infections in this context.**

This sentence now reads: “In 2020, Florida had the third greatest number of new HIV diagnoses in the United States with 3,258 new cases” (Page 4, lines 59-60).

3. **(Page 3, line 67) Define briefly and in simple terms “pre-exposure prophylaxis (PrEP)” and “post-exposure prophylaxis (PEP)”**

Information was added to explain PrEP and PEP in simpler terms: “Pre-exposure prophylaxis (PrEP) is a medication regimen recommended to prevent HIV among individuals who may be at increased risk of infection. Similarly, post-exposure prophylaxis is a treatment taken after a known or suspected HIV exposure” (page 4, lines 64-67).

4. **(Page 3, line 77-79) This sentence warrants citation “Many of these misconceptions can be traced back to the spread of misinformation during the AIDS epidemic in the 1980s, when little was known about the virus or its transmission.”**

Thank you for this suggestion. This sentence is now cited^1^ (pages 4-5, lines 77-78).

5. **(Page 4, line 79-83) Authors need to clarify the logic connected to how the misconceptions about HIV transmission could have the implications for community screening, care and ART adherence. For instance, how using public toilets, or mosquito bites could significantly discourage one from seeking community screening, pursing HIV care, and ART adherence. Does this mean, when people fear that casual contact could expose them to HIV, they may avoid testing and treatment? This may not always be the case, as some individuals might view these misconceptions as a health threat and still actively seek testing and treatment, motivated by the desire to safeguard their well-being despite their fears and false beliefs.**

Thank you for the suggestion. We edited the sentence and it now reads: “These false beliefs contribute to HIV-related stigma and decrease utilization of community screening, care establishment, and treatment adherence” (page 5, lines 81-83). Additionally, we added a source from the CDC which states that increased HIV stigma can discourage people from undergoing HIV testing.^2^

6. **(Page 4, Line 84-90) These sentences must be cited.**

Citations were added as follows: “Not only do transmission misconceptions limit patient utilization of HIV testing, but stigma also contributes to the limited use of preventative services.^3^ Stigma can be defined as personal, when people harbor negative beliefs or attitudes towards PLWH, or perceived, when people perceive a societal prejudice against PLWH but do not personally harbor negative opinions.^4^ In general, stigma is reinforced by societal structures and dependent on social interactions, witnessed acts of marginalization, misinformation, and discriminatory laws or policies^5”^ (page 5, lines 84-90).

7. **(Page 4, Line 92-93) Authors might need to clarify the exact nature of the relationship since it is not clear whether this relationship is positive (both types of stigma lead to increased care and adherence) or negative (high levels of stigma lead to decreased care and adherence to ART).**

Thank you. This sentence was edited to state: “Studies show that increased perceived and personal stigma are directly correlated with delayed later care utilization and decreased ART usage among PLWH” (page 5, line 114-115).

8. **(Page 5, line 99) Revise this phrase to best fit the context “These and other studies”.**

Thank you, this was updated (page 5, lines 92-93).

9. **(Page 5-6, Line 115-118) The aim is very bread and vague “This cross-sectional study aims to gather information about HIV education and perception in South Florida to improve community outreach efforts, and better understand how HIV knowledge and perception may differ by race, primary language, and country of origin. Suggested revision: “This cross-sectional study aims to assess HIV education and perceptions among diverse populations in South Florida to enhance public health community outreach efforts. Specifically, it will investigate how HIV knowledge and perceptions vary based on race, primary language, and country of origin.”**

Thank you for the suggestion—this has been edited to incorporate the suggested wording (page 6, lines 115-118).

**Material and methods:**

1. **(Page 6, line 120) Rephrase the following sentence for clarity “A 23-question survey was developed using questions adapted from previous validated surveys analyzing HIV knowledge and stigma among the public (S1 Appendix).” See suggested revision: “A survey instrument was developed consisting of 23 questions adapted from previously validated scales that measured HIV knowledge and stigma among the general public”.**

Thank you. The suggested revision was incorporated (page 6, lines 121-122).

2. **(Page 6, line 131-132) Back translation was performed to ensure the accuracy and cultural appropriateness of the translated materials? If yes, Authors should further elucidate this process and the No answer should be accompanied by explanation of how conceptual equivalence and content validity was ascertained on data collection instruments.**

Thank you. More information was added on how the accuracy of the translated materials was confirmed: “All surveys were translated from English to Spanish and Haitian Creole for administration in the participant’s native language in compliance with IRB standards (IRB#20220835), and then back translated to ensure accurate translation by a certified translator” (page 7, lines 129-132).

3. **(Page 6-7, line 120 -162) Authors should reorganize the manuscript under “Materials and methods” using level two subheadings for clarity and organization. Specifically, the following subheadings should be included:**

**1. Research Approach and Design: (Clearly define whether the study employed a quantitative approach using a cross-sectional design.)**

Thank you. The Materials and methods section was broken down by the 4 suggested subheadings and extensively edited. We more clearly specified that “This was a cross-sectional study utilizing a quantitative approach” (page 7, lines 124-125).

**2. Population, Sample Size, and Sampling: (Define the target population, explain how the sample size was determined using a specific formula with clear parameters, and state the sampling method used to select participants, justifying the choice of sampling method.)**

Thank you for this suggestion. We updated information about sample size calculations and the sampling method as follows: “Statistical power was assessed using Pass2020 with an alpha rate of .05. We examined power for sample sizes from 150 to 200, using Cohen’s w as the effect-size measure with a sample of 173. There is 80% power to uncover a chi-square with 1 degree of freedom with a w of .213. Cohen (1988) characterized a w=.10 as a small and a w=.30 as a medium effect, so this study has 80% power to uncover a small-to medium effect” (pages 7-8, lines 145-149).^6^

**3. Measures: (Describe and outline the items adapted from previously validated scales, report the internal consistency (Cronbach’s alpha coefficient) demonstrated by previous studies adopted the same scales, and include the internal consistency established by the current study.)**

Thank you for the suggestion. Questions were adapted from previously validated scales: “The HIV transmission knowledge questions were adapted from the Marcelin et al. and Herek et al. studies^7,8^ and the personal/perceived stigma questions were adapted from the STRIVE stigma questionnaire.^9^ The Herek et al. study, where the majority of transmission knowledge survey questions were derived, had a high internal consistency (α = .77-.79)” (page 8, lines 153-156). We calculated the internal consistency for our study by separately grouping the transmission knowledge, treatment knowledge, and stigma questions. This revealed Cronbach’s alpha coefficients of 0.47, 0.75. and 0.36 for transmission knowledge, treatment knowledge, and stigma respectively. We believe these numbers are low given that these question subgroups only contain 3-6 questions, which was intentional to prevent respondent fatigue. The goal of our research was to improve our understanding of patient education/ attitudes on numerous topics, and thus we would not anticipate a high internal consistency. We have focused on item-level analysis (and adequacy of knowledge) as now described in the methods (page 8, lines 156-157).

**4. Analysis: (Detail the statistical methods used to analyze the data, including any software utilized, the specific tests conducted, and the criteria for significance.)**

Thank you. This section more thoroughly describes the software utilized and tests performed: “Qualtrics was used to collect participant responses, and then the raw data was exported to Microsoft Excel. Response rates to each survey question were then aggregated in Excel according to the methods above and imported into GraphPad prism for statistical analysis. All data underwent chi-square analysis using 1 degree of freedom at an alpha level of 0.05” (page 9, lines 182-185).

**Results:**

1. **(Page 8, line 164) Rephrase “Among 173 total respondents” to “Out of 173 respondents,…” and change “ and” to “while” in the following sentence for proper context “149 received HIV testing and 24 declined HIV testing.”**

Thank you. These suggested edits were incorporated (page 9, line 187).

2. **(Page 8, line 166-171) all percentage values should be consistently placed within the parentheses for uniformity.**

This has been implemented (pages 9-10, lines 189-196). Thank you for the suggestion.

3. **(Page 8, line 165) Rephrase this part of the sentence for clarity “ranged in age from 18 to older than 75..” and change it to “…ranged between the age of 18 to over 75 years old,…”.**

This has been changed to the suggested wording (page 9, line 188).

4. **(Page 8, line 176), In accordance with PLOS One guidelines, figures should be referenced in the text as (Fig. 1, Fig. 2, Fig. 3, etc.) instead of using the term “figure.”**

Thank you for the suggestion. This was corrected throughout the manuscript.

5. **(Page 8-9, line 181-185) I strongly recommend authors to either discard this part or insert figure 1 as outlined and same comment applies to (Page 11, line 197 – 200) and (Page 12, line 213-215).**

Thank you for the suggestion. PLOS One formatting guidelines state, “Figure captions are inserted immediately after the first paragraph in which the figure is cited. Figure files are uploaded separately.”

6. **(Page 11, line 201 – 202) Incorporate all values from the chi-square test for statistical difference /independence among these categorical variables. Further, this comment applies to all reported results without chi-square statistic.**

Thank you for the suggestion. Every result reported in the results section now includes the degrees of freedom, sample size, chi square value, and p-value. In addition, we incorporated a new table (Table 2), which summarizes all the chi square results.

7. **(Page 10, line 186) As a general guideline, the title for tables should be placed above the table.**

Thank you. This was updated throughout the text.

8. **(Page 12, line 217) Rewrite “2-folds” as “two-folds” and this applies to (Page 14, Line 268)**

This was corrected.

9. **(Page 12, line 224) Rephrase “modified” to “influenced” throughout the manuscript.**

Thank you. This was corrected throughout the manuscript**.**

**Discussion:**

1. (**Page 12, line 226-227) This study’s aim indicates a significant discrepancy between what is outlined in the last sentence of the introduction and the actual aim (to gather information while also now is to identify knowledge gaps). Authors should consider incorporating a consistent aim throughout to remind readers of the clear research direction and scope. Use the suggested statement of the aim.**

Thank you—the sentence was revised as suggested (page 14, lines 255-258).

2. **(Page 12, line 227-229) Revise this sentence in accordance with the revision made above.**

This sentence was revised and now reads: “Through identification of these knowledge gaps, we aimed to improve education and outreach efforts to increase future utilization of HIV testing and prevention services among various demographic groups” (page 14, lines 258-260).

3. **(Page 12, line 230) Authors should not introduce new concepts in the discussion for consistency, I thought initially it was “HIV transmission” not “HIV risk”.**

The phrase was edited to “HIV transmission” (page 14, line 261).

4. **(Page 12-13, line 230-235) The discussion of the results should incorporate findings and outcomes from previous studies for comparison to provide context and highlight the value of the current research within the existing literature.**

Thank you for your suggestion. We have addressed how our findings relate to current research and added sources to the discussion: “While almost all participants understood that HIV can be spread through sex and injection drug use, many held additional misconceptions about how it is spread, and these misconceptions were more commonly held by Black and White Hispanic participants, compared to White non-Hispanic participants, reaffirming knowledge gaps demonstrated in prior studies.^10,11^ PrEP/PEP knowledge was more limited among White Hispanic participants, and our results demonstrate a clear need for improved educational efforts targeted at these groups which have lower rates of PrEP/PEP utilization.^12^**“** (page 14, lines 261-268).

**(Page 13, line 237) Write “about three thirds” instead of “Close to 100%” since this is a discussion section.**

This was corrected (page 14, line 270).

5. **(Page 13, line 249) Rephrase this part of the sentence for clarity “usage of PEP and PrEP have shown” to “the use of PEP and PrEP has proven to be significantly effective in reducing risks of HIV transmission” and cite the source (s).**

Thank you for the suggestion—this was updated and cited.^13^ (page 15, lines 282-283).

6. **(Page 14, line 256-259) Authors should clarify the connection between knowledge and language, as the study suggests that the lack of PEP and PrEP knowledge is somewhat influenced by language barriers. If the issue lies with language, it implies that the knowledge exists and is accessible but is hindered by challenges in decoding and interpreting it. Therefore, previous evidence should be cited to corroborate this assertion.**

Thank you for this suggestion. We believe that these results are influenced by language as well as cultural factors among Spanish-speaking patients: “These results are consistent with a past study indicating that Spanish-speaking Latino men had reduced PrEP awareness compared to their English-speaking counterparts.^14^ Furthermore, cultural distrust of medical care services and doctors within the Hispanic community likely contributes to these findings^15”^ (page 16, lines 297-300).

7. **(Page 14, line 261-263) This assertion should be supported by citations of scientific studies that have documented the connection between language and culture in the adoption of these preventive measures (PEP & PrEP) against HIV transmission.**

Thank you for this suggestion. We have included additional citations to better illustrate the effect of language and culture on preventive measures: “A qualitative study of Latino men who have sex with men found that there is skepticism about PrEP’s effectiveness specifically among monolingual Spanish speakers.^16^ Overall, we believe our data indicates that PrEP and PEP marketing companies should prioritize distributing translated and culturally sensitive materials to Hispanic communities in South Florida. In New York State, an enhanced PrEP Aware Week, utilizing social media messaging in both English and Spanish, increased PrEP prescription fills by 6-9%, indicating that culturally sensitive marketing can be incredibly effective”^17^ (page 16, lines 300-307).

8. **(Page 14, line 275) Authors should be careful not to use the term “screened positive” due to that stigma is typically measured using a validated scale rather than a screening tool. Various scales have been developed to assess different types of stigmas, such as HIV-related stigma, these scales include items that quantify the level of stigma an individual experiences or perceives, which makes it possible to measure the correlation between stigma and other variables. Thus, using a well-established stigma scale is not a limitation for measuring the correlation between stigma and other variables, as long as the scale is reliable and valid for the population being studied.**

Thank you for this suggestion we have removed the terms “screened positive” throughout the manuscript. Similarly, we have deleted the limitation regarding few patients reporting personal stigma.

9. **Under limitations, authors should address the following aspects:**
   a. **Design Used - The cross-sectional design adopted by the study**
   b. **Self-Report measures - The use of self-report measures may introduce bias due to social desirability.**
   c. **Sample size - The sample size may limit the generalizability of the findings, particularly if it is small.**

Thank you for the suggestion. We more thoroughly addressed these limitations in the discussion: “Furthermore, the cross-sectional nature of the study only allowed us to sample health fair participants at one timepoint, limiting our sample size and ability to draw conclusions about the impact of HIV education. The survey required patients to self-report measures, which may have introduced some bias due to social desirability” (page 17, lines 318-322).

**Conclusion:**

1. **(Page 15, line 286-287) “Stigma” is singular, so “was” should be used instead of “were.”**

This was corrected (page 17, line 331).

2. **(Page 15, line 287) “combat transmission” authors should specify that it “HIV transmission” for consistency.**

This was clarified (page 17, line 332).

**References:**

1. **(Page 17, reference 10) Authors should provide page number.**
2. **(Page 17, reference 12) Authors should provide an issue number for this source.**
3. **(Page 18, reference 21) Authors should provide an issue and page number for this source.**
4. **(Page 19, reference 28) Authors should provide a page number for this source.**

Thank you these have been corrected.

On the highest note: Additional recommendation:

1. **Authors should provide a table that presents the results from the Chi-Square test of difference, clearly outlining the variables compared, the chi-square statistic (χ²), degrees of freedom (df), sample sizes, and p-values to highlight significant and non-significant differences.**

Thank you for this suggestion. A table was added on page 12 with the requested variables.

**Additional Suggested Edits:**

**Wordiness & Redundancy**

**Some sentences are long and repetitive, making them harder to read.**

**Example:**

**"Many of these misconceptions can be traced back to the spread of misinformation during the AIDS epidemic in the 1980s, when little was known about the virus or its transmission."**

**Suggested Edit:**

**"Many misconceptions stem from misinformation spread during the 1980s AIDS epidemic when the virus was poorly understood."**

This was clarified (pages 4-5, lines 77-78).

**Passive Voice Usage**

**The manuscript frequently uses passive voice, which can make statements less direct and engaging.**

**Example:**

**"Survey data was analyzed using chi-square analysis at an alpha=0.05."**

**Suggested Edit:**

**"We analyzed survey data using chi-square tests with an alpha level of 0.05."**

Thank you. We updated this (page 2, line 38).

**Ambiguity in Some Statements**

**Example:**

**"Participants were classified as White non-Hispanic, White Hispanic, or Black as only two participants identified as Black Hispanic and were classified as mixed race."**

**Suggested Edit:**

**"Participants were categorized as White non-Hispanic, White Hispanic, or Black. Only two participants identified as Black Hispanic and were classified as mixed race."**

This suggested edit was updated (page 8, line 158-159).

**Brevity and Clarity:**

**Example:**

**Many of these misconceptions can be traced back to the spread of misinformation during the AIDS epidemic in the 1980s, when little was known about the virus or its transmission.**

**Suggested Edit:**

**Many misconceptions stem from misinformation spread during the 1980s AIDS epidemic when the virus was poorly understood.**

**Original:**

**Among 173 total respondents, 149 received HIV testing and 24 declined HIV testing.**

**Edited:**

**Of the 173 respondents, 149 underwent HIV testing, while 24 declined.**

These suggested edits were incorporated (page 2, line 39).

**Use “prevention” instead of “preventative” (more common in scientific writing).**

Thank you. This has been updated throughout.

**Introduce abbreviations when first mentioned and use them consistently i.e. PLWH**

Abbreviations are now included upon first mention.

**Reviewer #2:**

Thank you for your review. Your comments have greatly improved the manuscript.

1. **There is no clear sample size calculation provided. Was the sample size of 173 participants determined based on power analysis? Since participants were recruited from health fairs, they may already be more health-conscious than the general population. A brief discussion on potential selection bias would strengthen the methodology section.**

Thank you. We have since incorporated our power analysis reasoning for the sample size: “Statistical power was assessed using Pass2020 with an alpha rate of .05. We examined power for sample sizes from 150 to 200, using Cohen’s was the effect-size measure with a sample of 173. There is 80% power to uncover a chi-square with 1 degree of freedom with a w of .213. Cohen (1988) characterized a w=.10 as a small and a w=.30 as a medium effect, so this study has 80% power to uncover a small to medium effect” (page 7-8, lines 145-149). A sentence was added in the discussion to acknowledge the selection bias introduced by collecting responses at health fairs: “Additionally, participants were likely inherently health conscious as they were sampled from preventative health fairs, which also promoted a selection bias” (page 17, lines 324-325).

2. **The study considers race, language, and country of origin, but educational attainment could also significantly impact HIV knowledge. Were potential confounders controlled for?**

Thank you for the clarification. Yes, we broke down each race and language groups by education level and found that there was no significant difference among the groups of education levels: “Education level of participants was also taken into consideration to account for this as potential confounder; however, there was no significant difference in the rates of college educated individuals broken down by race or language” (page 13, lines 237-240).

3. **The study suggests that interventions should target Hispanic communities, but the data primarily focuses on White Hispanic participants. Can these findings be generalized to all Hispanic groups, or are they specific to this subgroup?**

Thank you for your question. Our findings are specific to White Hispanic individuals residing in South Florida. While we had a wide variety of Hispanic patients born in many countries across North America, Central America, South America, and the Caribbean, we only had two patients who identified as both Black and Hispanic and they were classified as “mixed race”. This information was added to the Materials and methods section: “Participants were categorized as White non-Hispanic, White Hispanic, or Black. Only two participants identified as Black Hispanic and were classified as mixed race. All participants that we describe as Black are non-Hispanic." (page 8, lines 158-160).

**Reviewer #3:**

Thank you for your review. Your comments have greatly improved the manuscript.

1. **Please provide the inclusion and exclusion criteria you used in recruiting survey respondents.**

Thank you for your comment. We included more detailed information about our inclusion/exclusion criteria: “We included health fair participants over the age of 18 capable of providing informed consent. No participants who completed the survey were excluded from analysis” (page 7, lines 143-145).

2. **Among the respondents who consented to HIV screening, how many percent tested reactive, non-reactive? Perhaps it would be significant to assess their test results against their HIV transmission knowledge.**

Thank you for your suggestion. Among those who received HIV screening, we did not have any reactive tests, and thus we did not analyze this information.

3. **In Table 1, please include the key population in which the respondents qualify as (MSM, PWID, TGW etc).**

Unfortunately, these questions were not included in the survey and thus cannot be analyzed.

**Works Cited**

1.	Heller J. Rumors and Realities: Making Sense of HIV/AIDS Conspiracy Narratives and Contemporary Legends. *Am J Public Health*. 2015;105(1):e43. doi:10.2105/AJPH.2014.302284

2.	Stigma and HIV | HIV | CDC. Accessed April 1, 2025. https://www.cdc.gov/hiv/health-equity/index.html

3.	Babel RA, Wang P, Alessi EJ, Raymond HF, Wei C. Stigma, HIV Risk, and Access to HIV Prevention and Treatment Services Among Men Who have Sex with Men (MSM) in the United States: A Scoping Review. *AIDS Behav*. 2021;25(11):3574-3604. doi:10.1007/S10461-021-03262-4/TABLES/2

4.	Pedersen ER, Paves AP. Comparing perceived public stigma and personal stigma of mental health treatment seeking in a young adult sample. *Psychiatry Res*. 2014;219(1):143. doi:10.1016/J.PSYCHRES.2014.05.017

5.	Bolster-Foucault C, Fane BHM, Blair A. Structural determinants of stigma across health and social conditions: a rapid review and conceptual framework to guide future research and intervention. *Health Promot Chronic Dis Prev Can*. 2021;41(3):85. doi:10.24095/HPCDP.41.3.03

6.	Cohen J. Statistical power analysis for the behavioral sciences. Published online 1988. Accessed April 20, 2025. https://docs.opendeved.net/lib/9UDZ3UVQ

7.	Herns Marcelin L, McCoy HV, DiClemente RJ. HIV/AIDS Knowledge and Beliefs Among Haitian Adolescents in Miami-Dade County, Florida. *J HIV AIDS Prev Child Youth*. 2006;7(1):121. doi:10.1300/J499V07N01_07

8.	Herek GM, Capitanio JP, Widaman KF. HIV-Related Stigma and Knowledge in the United States: Prevalence and Trends, 1991–1999. *Am J Public Health*. 2002;92(3):371. doi:10.2105/AJPH.92.3.371

9.	Stangl A, Brady L, Fritz K. *Measuring HIV Stigma and Discrimination*.; 2012. Accessed April 21, 2025. https://strive.lshtm.ac.uk/system/files/attachments/STRIVE%20stigma%20measurement.pdf

10.	Kenya S, Okoro I, Wallace K, Carrasquillo O, Prado G. Strategies to Improve HIV Testing in African Americans. *J Assoc Nurses AIDS Care*. 2015;26(4):357. doi:10.1016/J.JANA.2015.04.001

11.	Cianelli R, Villegas N, Gonzalez-Guarda R, Kaelber L, Peragallo N. HIV Susceptibility Among Hispanic Women in South Florida. *J Community Health Nurs*. 2010;27(4):207. doi:10.1080/07370016.2010.515458

12.	Campbell JT, Adams OR, Bennett-Brown M, Woodward B, Gesselman AN, Carter G. PrEP Familiarity, Interest, and Usage Among 364 Black and Hispanic Adults in Indiana. *Front Public Health*. 2022;10:810042. doi:10.3389/FPUBH.2022.810042/BIBTEX

13.	HIV: PrEP and PEP. Accessed April 20, 2025. https://medlineplus.gov/hivprepandpep.html

14.	Brooks RA, Nieto O, Landrian A, Donohoe TJ. Persistent stigmatizing and negative perceptions of pre-exposure prophylaxis (PrEP) users: implications for PrEP adoption among Latino men who have sex with men. *AIDS Care*. 2019;31(4):427-435. doi:10.1080/09540121.2018.1499864

15.	Dolwick Grieb SM, Desir F, Flores-Miller A, Page K. Qualitative assessment of HIV prevention challenges and opportunities among Latino immigrant men in a new receiving city. *J Immigr Minor Health*. 2015;17(1):118-124. doi:10.1007/S10903-013-9932-3

16.	Brooks RA, Nieto O, Landrian A, Donohoe TJ. Persistent Stigmatizing and Negative Perceptions of Pre-Exposure Prophylaxis (PrEP) Users: Implications for PrEP Adoption among Latino Men Who Have Sex with Men. *AIDS Care*. 2018;31(4):427. doi:10.1080/09540121.2018.1499864

17.	Tesoriero JM, Yuan Y, Newport R, et al. Assessing the Impact of a PrEP Aware Week Campaign on PrEP Prescription Fills in NYS. *Journal of Public Health Management and Practice*. 2023;28(5):463. doi:10.1097/PHH.0000000000001554
