# Supplementary material for: HIV knowledge and reported stigma among South Florida health fair participants
Source: PLoS One. 2025 Aug 19;20(8):e0329665. doi: 10.1371/journal.pone.0329665 (PMC12364354; doi:10.1371/journal.pone.0329665)
Supplement: Attachment — Submitted filename: Response HIV_7.6_ (1).docx [file pone.0329665.s005.docx]

**Reviewer Responses**

Thank you for reviewing. Your comments have greatly improved the manuscript.

Reviewer 1
**Line 44–49, Page 3:

- “Transmission knowledge was significantly influenced by race” and “Familiarity with PrEP and/or PEP was also influenced by race…”.
- The use of “influenced” implies a causal relationship, which is not supported by a cross-sectional design and Chi-square test employed.
- Gold standard way of reporting, which reflects what Chi-square actually tests “Familiarity with PrEP and/or PEP was significantly associated with race…”**

Thank you for this suggestion. The wording has been corrected throughout the manuscript so that “influenced” was replaced with “associated with” to better describe the non-causal relationship (lines 45, 48, 329, 361, and 379).

**Line 217, Page 12 and throughout results section:

- Some chi-square statistics use more than two decimal places including for p-values.
- I strongly suggest consistency in rounding to two for chi-square statistic and three decimals for p-values throughout the manuscript.**

Thank you for this suggestion. Rounding is now consistent throughout the manuscript to two decimals for chi-square statistics and three decimals for p-values.

**Line 151-179, Page 8-9, Measures:

- The measures section should use consistent tense and structured subheadings
- Present each construct separately and clearly with the name and description of the instrument employed to measure the construct. For example:
HIV Transmission Knowledge and HIV stigma are the main constructs measured using previously validated instruments.
- Describe the origin of items, psychometric properties, scoring, and interpretation.
- Clearly state how variables were operationalized and analysed.
- There is no mention of Cronbach’s alpha coefficient for the adapted versions in current study. Given that the instruments were translated and adapted, I recommend that the authors either include a table summarizing the internal consistency (e.g., Cronbach’s alpha) for each scale or report these values directly under the Measures section to ensure transparency on the reliability of the instruments.**

Thank you for your comments. Subsection headings were added, and we updated verb tense throughout the section. We updated information to better describe the origin of questionnaire items: “HIV transmission knowledge and HIV stigma are the main constructs measured using previously validated instruments. One third of the HIV transmission knowledge questions are adapted from the Marcelin et al. study and two thirds of the transmission knowledge questions are derived from the Herek et al. study. All of the personal/perceived stigma questions are adapted from the STRIVE stigma questionnaire. The Herek et al. study, has a high internal consistency (α = .77-.79) but there are no reported internal consistencies reported for the Marcelin et al. or STRIVE stigma questionnaires” (page 8, lines 188-194).

A new subsection was incorporated to better define survey scoring; “Transmission knowledge scores are classified as adequate if participants missed no more than one question out of the six total. This cut off was determined based on pilot data from the initial health fair survey responses (n=12), which demonstrated a mean of 80% correct. Participants are considered familiar with PrEP/PEP if they noted familiarity with either medication on the questionnaire. For the four stigma questions, participants demonstrated personal stigma if they screened positive on one of the first two questions or perceived stigma if they screened positive on at least one of the last two questions” (page 9, lines 274-280).

Thank you for your recommendation to report the Cronbach’s alpha coefficient for our adapted survey. We included this information in the measures section to ensure transparency of the reliability of the survey: “We calculated the internal consistency of our study by performing Cronbach’s alpha analysis on PEP/PrEP treatment knowledge questions and calculated alpha coefficients of 0.75.” (page 10, lines 308-311). We did not report an alpha for HIV knowledge because our survey included true-false statements and the number correct is a clear indicator of a person’s knowledge of the topic. The 2-item stigma scales had a low alpha value and thus did not make a reliable scale. Therefore, we removed the chi-square findings and acknowledged this limitation in the results: “Perceived and personal stigma had a low Cronbach’s alpha coefficient value and thus we drew limited conclusions about their association with race, country of origin, and primary language” (page 15, lines 261-262)

**Line 167–169, Page 8-9:

- Defining adequate HIV transmission knowledge as missing ≤1 question is an arbitrary without statistical rationale.
- Provide justification from prior literature or explain how pilot data support this threshold as valid (acknowledge this as a limitation).**

Thank you for this suggestion. The rationale for determining the cut-off was determined from pilot data: “Transmission knowledge scores are classified as adequate if participants missed no more than one question out of the six total. This cut off was determined based on pilot data from the initial health fair survey responses (n=12), which demonstrated a mean of 80% correct” (page 9, lines 274-276). Information about this limitation was added to the discussion section: “When evaluating survey responses, we utilized pilot data to determine the cut-off for sufficient transmission knowledge among the population which introduces variability” (page 18, lines 490-492).

**Line 237–240, Page 13:

- Authors claim that “there was no significant difference in rates of college educated individuals broken down by race or language” yet no statistical test or supporting data.
- Provide chi-square results that support this claim
- The authors mention race and language when exploring potential confounders, yet none of the central variables (HIV knowledge, stigma, PrEP/PEP awareness) were included in this analysis, making the confounder exploration to be methodologically unsound.**
Thank you for these comments. The manuscript was updated to include the non-significant chi-square statistics: “There was no significant difference in the rates of college educated individuals broken down by race (χ²(2, N = 116)= 3.85, p =.146), language (χ²(1, N = 97)= 0.01, p =.938), or country of origin (χ²(1, N = 98)= 0.68, p =.408)” (page 14, lines 371-373). We removed the statement about exploring potential confounders.

**Figure 3 and Lines 231–233, Page 13:

- Some subgroup analyses, such as bilingual or Creole speakers, include very small sample sizes (n=9–11).
- Concern is that these small groups may produce unstable estimates and wide margins of error, which may invalidate statistical inferences.
- Acknowledge this limitation explicitly in the Discussion and temper your conclusion based on these groups.**

Thank you for this suggestion. We did not draw conclusions about bilingual or Creole speakers given the small sample sizes. This limitation was added to the discussion section: “Moreover, we could not adequately assess bilingual or Haitian-Creole speakers given the low sample size” (page 18, lines 485-486).

**Line 251-252, Page 14:**

**- Authors mentioned that “Otherwise, perceived stigma was not significantly influenced by race, country of origin, or primary language.” Yet without chi-square statistics.
Absence of adjusted analysis:
- All findings rely solely on bivariate chi-square analyses.
- Bivariate associations do not account for confounding exploration mentioned in the manuscript.
- Consider conducting a multivariate logistic regression for key outcomes or justify the decision not to do so in the limitations.**

Thank you for the suggestions. The chi-square statistics are now reported in the manuscript: “Otherwise, perceived stigma was not significantly associated with race (χ²(2, N = 163)= .56, p =.757), country of origin (χ²(1, N = 137)= 1.76, p =.185), or primary language (χ²(1, N = 138)= .62, p =.432)” ( page 15, line 399-401). We justified our reasons for running chi-square analysis rather than a multivariable logistic regression in the discussion section: “While we assessed interactions via bi-variate chi-square analysis, we did not run a multivariable logistic regression to assess confounders because numerous demographic variables including race, country of origin, and language are interrelated and cannot be considered independent variables” (page 18, lines 493-496).

Minor concerns:
**- Recommendation for language editing.
- Line 183 -184, page 9 – “All data underwent chi-square analysis…” avoid using passive voice instead of active voice. “All data were analysed using chi-square…”
- Use of “unfamiliarity” consider using “lack of familiarity”**

Thank you. This has been corrected. (lines 54, 350, 367).

**Line 345 and 351, Page 19:

- Reference 4 (Duplicate of Ref. 1):
- “Statistics Overview | Statistics Center | HIV/AIDS | CDC. Accessed April 15, 2024.**[**https://www.cdc.gov/hiv/statistics/overview/index.html**](https://urldefense.com/v3/__https:/www.cdc.gov/hiv/statistics/overview/index.html__;!!KVu0SnhVq1hAFvslES2Y!OjOOYb5eLenr0PSyKRYNA3B3Ec1-AkOINpKXG34sddZMbM5yV6pFi8aQLR4odUJFYm7yWi3WIRnvnDeAEQ$)**”**

Thank you. The duplicate citation has been deleted from the manuscript.
